# Activation of Toll Immune Pathway in an Insect Vector Induced by a Plant Virus

**DOI:** 10.3389/fimmu.2020.613957

**Published:** 2021-01-08

**Authors:** Yu-Juan He, Gang Lu, Yu-Hua Qi, Yan Zhang, Xiao-Di Zhang, Hai-Jian Huang, Ji-Chong Zhuo, Zong-Tao Sun, Fei Yan, Jian-Ping Chen, Chuan-Xi Zhang, Jun-Min Li

**Affiliations:** ^1^ College of Plant Protection, Nanjing Agricultural University, Nanjing, China; ^2^ State Key Laboratory for Managing Biotic and Chemical Threats to the Quality and Safety of Agro-products, Key Laboratory of Biotechnology in Plant Protection of MOA of China and Zhejiang Province, Institute of Plant Virology, Ningbo University, Ningbo, China

**Keywords:** Toll pathway, rice stripe virus, small brown planthopper, immune perception, protein interaction

## Abstract

The Toll pathway plays an important role in defense against infection of various pathogenic microorganisms, including viruses. However, current understanding of Toll pathway was mainly restricted in mammal and some model insects such as *Drosophila* and mosquitoes. Whether plant viruses can also activate the Toll signaling pathway in vector insects is still unknown. In this study, using rice stripe virus (RSV) and its insect vector (small brown planthopper, *Laodelphax striatellus*) as a model, we found that the Toll pathway was activated upon RSV infection. In comparison of viruliferous and non-viruliferous planthoppers, we found that four Toll pathway core genes (*Toll*, *Tube*, *MyD88*, and *Dorsal*) were upregulated in viruliferous planthoppers. When the planthoppers infected with RSV, the expressions of *Toll* and *MyD88* were rapidly upregulated at the early stage (1 and 3 days post-infection), whereas *Dorsal* was upregulated at the late stage (9 days post-infection). Furthermore, induction of Toll pathway was initiated by interaction between a Toll receptor and RSV nucleocapsid protein (NP). Knockdown of *Toll* increased the proliferation of RSV in vector insect, and the ds*Toll*-treated insects exhibited higher mortality than that of ds*GFP*-treated ones. Our results provide the first evidence that the Toll signaling pathway of an insect vector is potentially activated through the direct interaction between Toll receptor and a protein encoded by a plant virus, indicating that Toll immune pathway is an important strategy against plant virus infection in an insect vector.

## Introduction

In invertebrates, host defense against pathogens, including bacteria, fungi, and viruses, is known to rely on innate immunity, while in vertebrates, the innate immune system provides the first defense line against pathogens before activation of acquired immune response (1). In insects, various evolutionarily conserved signaling pathways mediate antiviral immunity, including small RNA interference (RNAi), Toll, the immune deficiency (IMD), and JAK-STAT (2, 3). These pathways mainly rely on different pattern recognition receptors (PRRs), which recognize signature molecules of pathogens, known as pathogen associated molecular patterns (PAMPs) and induce downstream effectors against viral infection (4, 5). Toll receptor superfamily, including invertebrate Tolls and vertebrate Toll-like receptors (TLRs), is important class of PRRs and the primary sensor of pathogens in all metazoans (6). The activation of Toll pathway in vertebrate is initiated by TLRs binding to various PAMPs, whereas in invertebrate, it is activated indirectly by Toll receptors binding to the cytokine-like molecule Spätzle (Spz) (7). Tolls and TLRs are characterized by an extracellular domain containing leucine-rich repeats (LRRs), a transmembrane domain, and a cytoplasmic tail that contains a conserved region called the Toll/IL-1 receptor (TIR) domain (8). The first identified Toll (Toll1) is the receptor of the Toll pathway, and to date, nine Toll genes have been identified in *Drosophila* (8). In invertebrate, pathogen infection is censored by extracellular recognition and the inactive precursor of the Spz is cleaved to active form. Then the activated Spz binds to Toll receptor and a cassette of proteins (MyD88, Tube and Pelle) are recruited to assemble a receptor-proximal oligomeric complex (9–11). In *Drosophila*, the complex further trigger the phosphorylation and degradation of Cactus, freeing Dorsal or Dif (Dorsal-related immunity factor) to transfer from the cytoplasm into the nucleus for the regulation of different antibacterial peptides (AMPs) expressions (12).

Although the importance of the Toll pathway against bacteria and fungi has been well demonstrated, accumulated evidences suggested that it also plays essential antiviral roles in invertebrate, such as the fly (*Drosophila*) and mosquitoes (*Culex*, *Aedes*, and *Anopheles*) (1, 13). The importance of Toll pathway against virus was firstly reported in *Drosophila* when challenged with *Drosophila* X virus (DXV) infection (1). Further studies indicated that the Toll pathway also mediate resistance to other RNA viruses including *Drosophila* C virus, cricket paralysis virus, flock house virus, and norovirus (14). In mosquito, Toll immune pathway was activated upon viral infection, and they controlled the conserved anti-dengue defenses across diverse *Aegypti* strains and against multiple dengue virus serotypes (13, 15). Interestingly, recent studies found that several members of Toll receptors can also act as PRRs analogous to the TLRs in mammal, triggering conventional or non-conventional Toll-Dorsal pathway. RNAi screening suggested that Toll-4 might be one of upstream PRR to detect white spot syndrome virus (WSSV) infection in shrimp, and thereby leading to conventional Toll-Dorsal pathway (16). Another example is three shrimp Tolls (Toll1-3) directly bind to PAMPs from bacterial infection, resulting in Dorsal translocation into nucleus to regulate the expression of different AMPs (17). In contrast, *Drosophila* Toll-7 can also act as a PRR and directly interact with vesicular stomatitis virus (VSV) at the plasma membrane, but induces antiviral autophagy independent of the canonical Toll-Dorsal signaling pathway (2).

Rice stripe virus (RSV) is a filamentous, negative-strand RNA virus of the genus *Tenuivirus* that causes rice stripe disease, one of the most severe rice diseases in East Asia (18–20). RSV is transmitted by the vector insect, small brown planthopper (SBPH, *Laodelphax striatellus*), in a persistent-propagative manner. RSV can replicate in *L. striatellus*, and can be transmitted to the progeny of the planthopper through infection of the embryos or germ cells in the female insects (21). The viral genome of RSV consists of four single-stranded RNA segments: RNA1-RNA4. RNA1 is negative-sense RNA and encodes a 337-kDa protein referred to as RNA-dependent RNA polymerase (22). The other three genomic segments exhibit ambisense coding features and each RNA encodes two proteins. Sense and antisense strands of RNA2 encode RNA silencing suppressor NS2 and the putative membrane glycoprotein NSvc2, respectively (23–25). RNA3 encodes a second viral suppressor NS3 (26), and complementary sense RNA3 (vcRNA3) encodes the nucleocapsid protein (NP) (27, 28). RNA4 encodes the disease-specific protein NS4 (29), and vcRNA4 encodes the movement protein (MP) (30, 31). Previous studies suggested the induced active response of *L. striatellus* during RSV infection. For example, analysis of viral-derived small interfering RNAs (siRNAs) revealed that RNAi-mediated antiviral response can successfully be induced by the infection of RSV and another reovirus, rice black-streaked dwarf virus (32). Activation of c-Jun N-terminal kinase (JNK) promoted RSV replication in *L. striatellus*, whereas JNK inhibition caused a significant reduction in virus production and thus delayed disease incidence in plants (33). In addition, silencing of the autophagy-related-8 (*Atg8*) expression of *L. striatellus* significantly decreased the phosphorylation of JNK in the midgut of the planthoppers, suggesting that ATG8 might activate the JNK machinery (34). Nevertheless, to date, whether the classical Toll-Dorsal pathway involved in antiviral response of *L. striatellus* or other plant virus vectors have never been investigated.

In this study, open reading frames (ORFs) of four core components from Toll pathway, including *Toll*, *Tube*, *MyD88*, and *Dorsal*, were identified from *L. striatellus* and their potential antiviral roles were further explored. Our results revealed that the Toll signaling pathway in *L. striatellus* is potentially induced through the direct interaction between Toll and RSV-NP. Knockdown of Toll increased the replication of RSV, indicating that Toll in insect vectors might act as PRR in perceiving plant viruses, similar to that of TLRs in mammalian.

## Materials and Methods

### Insects

The planthopper populations are maintained on susceptive japonica rice seedlings (cv Wuyujing No. 3) in a temperature-controlled room at 25 ± 1°C, with 70–80% relative humidity, and a light/dark photoperiod of 14/10 h. The infection ratio of the viruliferous planthopper population (RSV-infected) was around 80% and monitored every 3–4 generations by reverse transcription polymerase chain reaction (RT-PCR) as described previously (32).

### Gene Cloning and Phylogenetic Analysis

Four Toll pathway core genes (*Toll*, *Tube*, *MyD88*, and *Dorsal*) were obtained from transcriptome of *L. striatellus* (Accession Number: SRR4020768) by homology search based on the corresponding genes of *Nilaparvata lugens* as query sequences (nlToll, XP_022198839; nlTube, XP_022207725; nlMyD88, XP_022187892; nlDorsal, XP_022195378). Conserved protein domains were predicted using NCBI conserved domains database (https://www.ncbi.nlm.nih.gov/Structure/cdd/wrpsb.cgi). Phylogenetic trees were constructed based on the deduced amino-acid sequences in MEGA 6.0 using the maximum likelihood (ML) algorithm with 1,000 bootstrap replications. The full-length ORFs of the four identified genes were amplified with the respective primer pairs (**Supplementary Table 1**) from planthoppers using RT-PCR and further confirmed by Sanger sequencing (Sangon, China).

### Yeast Two-Hybrid Assay

For the yeast two-hybrid assay (Y2H) interaction assay, the full-length of RSV NS2, NSvc2-C (C-terminal of glycoprotein), NSvc2-N (N-terminal of glycoprotein), NS3, NP, NS4, and MP were cloned into the DNA-binding domain of the vector pGBK-T7 to create bait plasmids. The full-length ORF of *Toll* was cloned into the activation domain of the yeast vector pGAD-T7. Yeast cells (AH109) were co-transformed with RSV protein libraries and pGAD-T7-*Toll*. Positive clones were selected on quadruple dropout medium (SD/-Leu/-Trp/-His/-Ade).

### Bimolecular Fluorescence Complementation Assays

To further confirm the protein interactions, the full-length genes of RSV proteins NS2, NSvc2-C, NSvc2-N, NS3, NP, NS4, and MP were amplified and cloned into pCV-nYFP expression vector, respectively. The full-length ORF of *Toll* was cloned into pCV-cYFP expression vector. Constructed vector pCV-cYFP-*Toll* were then transformed into *Agrobacterium tumefaciens* GV3101 by heat transfer method, and co-transformed with pCV-nYFP-*NS2*, pCV-nYFP-*NSvc2-C*, pCV-nYFP-*NSvc2-N*, pCV-nYFP-*NS3*, pCV-nYFP-*NP*, pCV-nYFP-*NS4*, and pCV-nYFP-*MP*, into *Nicotiana benthamiana*, respectively. YFP fluorescence signal was observed under Nikon confocal (Nikon, Japan).

### Total RNA Extraction and Quantitative Real-Time PCR

The total RNA was extracted using TRIzol (Invitrogen, USA) according to the manufacturer’s instructions. The concentration and quality of total RNA was determined using a NanoDrop spectrophotometer (Thermo Scientific, USA). The first strand of complementary DNA (cDNA) from 1,000 ng of total RNA was synthesized with HiScript ^®^II Q RT SuperMix for qPCR (+gDNA wiper) (Vazyme, China) following the manufacturer’s protocol. In brief, quantitative real-time PCR (qPCR) was performed in 10 µl-reaction agent containing 0.5 µl of template cDNA and 5 µl of Hieff ^®^ qPCR SYBR Green PCR Master Mix (YESEN, China), 0.2 µl of 1 µM forward and reverse primers, and 4.1 µl of ddH_2_O on LightCycler^®^ 480 II (Roche, Switzerland). The thermal cycling conditions were 95°C for 5 min, 40 cycles of 95°C for 30 s, 60°C for 30 s, and 70°C for 30 s, followed by melting curve analysis. The data were analyzed using the 2^−ΔΔCT^ method and statistically significant differences at *P* < 0.05 (*) and *P* < 0.01 (**) level are indicated according to one-way analysis of variance (ANOVA) test.

### Expression Profiles of the Four Toll Pathway Genes

Planthopper samples of different developmental stages (eggs, 1^st^ to 5^th^ instar nymphs, female adults, and male adults) and various tissues (salivary gland, gut, ovary of female adult, epidermis, hemolymph, fat body, and testis of male adult) from non-viruliferous *L. striatellus* were collected. For the collection of hemolymph and fat body, the PBS solution after the dissect of planthoppers was centrifuged at 5,000 × *g* for 5 min at 4°C, and the hemolymph in supernatant and fat body in precipitate were separately collected, respectively. Five independent replicates were used in this experiment. For the collection of different developmental stages, various numbers of insects were obtained according to the sample size for each replicate. While for tissues, each replicate contains different tissues derived from approximately 40–50 individual adult planthoppers.

To determine the expression of *Toll*, *Tube*, *MyD88*, and *Dorsal* in response to RSV infection, approximately 20 adult planthoppers from non-viruliferous and viruliferous cultures were collected for RNA extraction individually. In addition, to further investigate the response of Toll pathway core gene expressions during the whole process of RSV infection, approximately 1,000 2^nd^ instar nymphs of non-viruliferous planthoppers were transferred and feeding onto RSV-infected rice seedlings for 2 days. Then the planthoppers were transferred to healthy rice seedlings and collected at various time points (1, 3, 6, 9, or 12 days post-infection). About 20 planthoppers were collected at each time point and expressions of Toll pathway genes were determined from individual insect by qPCR as described above.

### Double-Stranded RNA Synthesis and Delivery

*Toll*, *Tube*, *MyD88*, and *Dorsal* fragments of *L. striatellus* were amplified using gene-specific primer ligated with a T7-promoter sequence, and the green fluorescent proteins (*GFP*) fragment was used as a negative control. The primers used for the amplification were listed in **Supplementary Table 1**. The double-stranded RNA (dsRNA) was synthesized using the T7 RiboMAX Express RNAi System (Promega, USA) following the manufacturer’s instructions. The quality of synthesized dsRNA was evaluated using agarose gel electrophoresis. Each planthopper was injected with 40 nl of dsRNA into the insect ventral thorax with a glass needle (35).

### Quantification of Rice Stripe Virus Proliferation

Adult planthoppers from viruliferous culture were collected and injected with ds*Toll*, ds*Tube*, ds*MyD88*, and ds*Dorsal*, respectively. ds*GFP* was used as a negative control. Each of the injected planthopper was used for RNA extraction and the RNAi efficiency was determined at 48 h post-injection using qPCR. The accumulation level of RSV was quantified by qPCR as described above with specific primer pairs for RSV-NP.

### Accumulation of Rice Stripe Virus and Mortality of Planthoppers During Rice Stripe Virus Infection

Second instar planthoppers from non-viruliferous culture were injected with ds*Toll* individually and maintained in healthy rice seedling for 2 days. The injected planthoppers were then transferred onto RSV-infected rice seedlings for another 2 days for virus acquisition. Finally, the planthoppers were moved to healthy rice seedling again and collected at various time points for the detection of virus accumulation. ds*GFP* was used as a negative control. Each of the injected planthopper was used for RNA extraction at 0, 1, 3, 9 days post-RSV acquisition. The expression of RSV-NP was measured by qPCR after silencing of *Toll*. Approximately 20 planthoppers were used for the detection at each time point. Meanwhile, the mortality rate of ds*Toll* injected planthoppers was investigated. Three biological replicates were performed for each treatment in this experiment.

## Results

### Identification of the Toll Pathway Core Genes in *Laodelphax striatellus*

**Figure 1:**
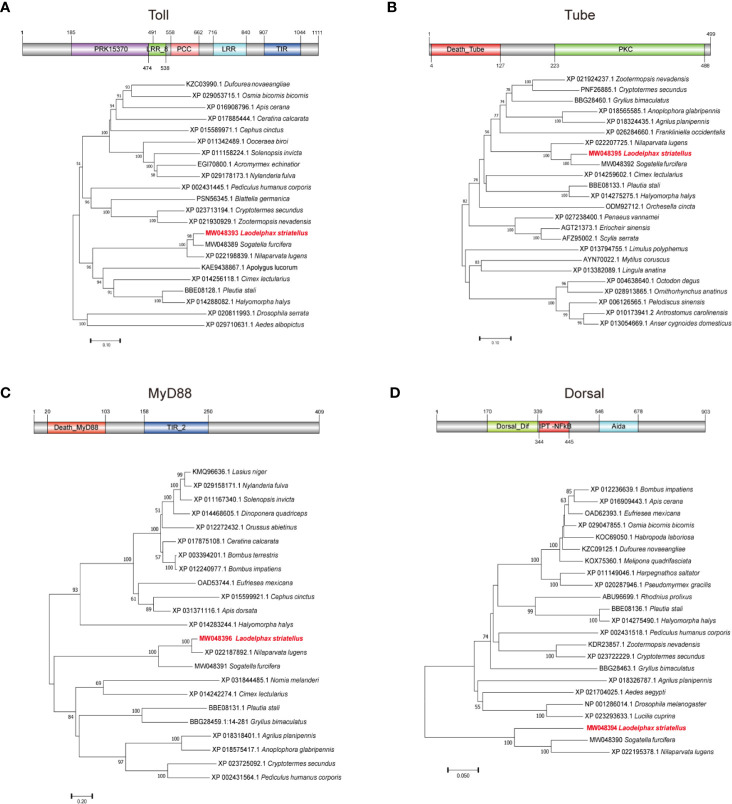
The architecture and phylogenetic analysis of *Toll*, *Tube*, *MyD88*, and *Dorsal*. **(A)** *Toll* contains five conserved domains: PRK15370, TIR, LRR_8, leucine-rich repeat (LRR), and PCC. **(B)** *Tube* contains two conserved domains: Death and S_TKC. **(C)** *MyD88* contains two conserved domains: Death_MyD88 and TIR_2. **(D)** *Dorsal* contains three conserved domains: Dorsal_Dif, RHD_dimer, and AidA. Phylogenetic tree analysis with the maximum likelihood method was based on homologous amino-acid sequences of *Laodelphax striatellus* and other insects.

To explore the potential antiviral roles of classic Toll pathway in *L. striatellus*, full ORF of Toll pathway core genes (*Toll*, *Tube*, *MyD88*, and *Dorsal*) were identiﬁed and cloned. The ORF of *Toll* consists of 3336 bp nucleotides encoding a predicted protein of 1,111 amino-acid residues with a calculated molecular mass of 125.82 kDa. The predicted Toll protein contains five conserved domains including a PRK15370 super family (type III secretion system effector E3 ubiquitin transferase SlrP), a LRR_8 (Leucine rich repeat), a PCC super family (polycystin cation channel protein), a LRR, and a TIR (Toll—interleukin 1—resistance) (**Figure 1A**); *Tube* contains a 1,500 bp ORF, encoding a predicted protein of 499 amino-acid residues with a calculated molecular mass of 55.58 kDa. The putative Tube protein contains a Death_Tube domain and a PKc (protein kinases) domain (**Figure 1B**); *MyD88* consists of 1,230 bp and encodes a predicted protein of 409 amino acids. The putative MyD88 protein contains two conserved domains Death_MyD88 and TIR_2 (a family of bacterial TLRs) (**Figure 1C**). *Dorsal* contains a continuous 2,712 bp ORF, encoding a predicted protein of 903 amino-acid residues. The putative Dorsal protein contains domains including a Dorsal_Dif, a RHD_dimer (Rel homology dimerization), and an AidA superfamily (**Figure 1D**). The full ORF sequences of *Toll*, *Tube*, *MyD88*, and *Dorsal* were submitted to GenBank with the accession numbers of MW048393, MW048395, MW048396, and MW048394.

Homology analysis showed that the predicted amino acids of the four Toll pathway proteins of *L. striatellus* share highest homologies to the other two rice planthoppers, *N. lugens* and *Sogatella furcifera*, with identities of 88.44 and 88.07% for Toll, 70.18 and 60.39% for Tube, 77.64 and 31.05% for MyD88, 65.11 and 49.77% for Dorsal, respectively. Phylogenetic analysis based on the putative amino-acid sequences suggested the four proteins of *L. striatellus* clustered together with the other two planthoppers (*N. lugens* and *S. furcifera*) with high strap value support (**Figure 1**).

### Interaction Between *Laodelphax striatellus* Toll and Rice Stripe Virus-NP

**Figure 2:**
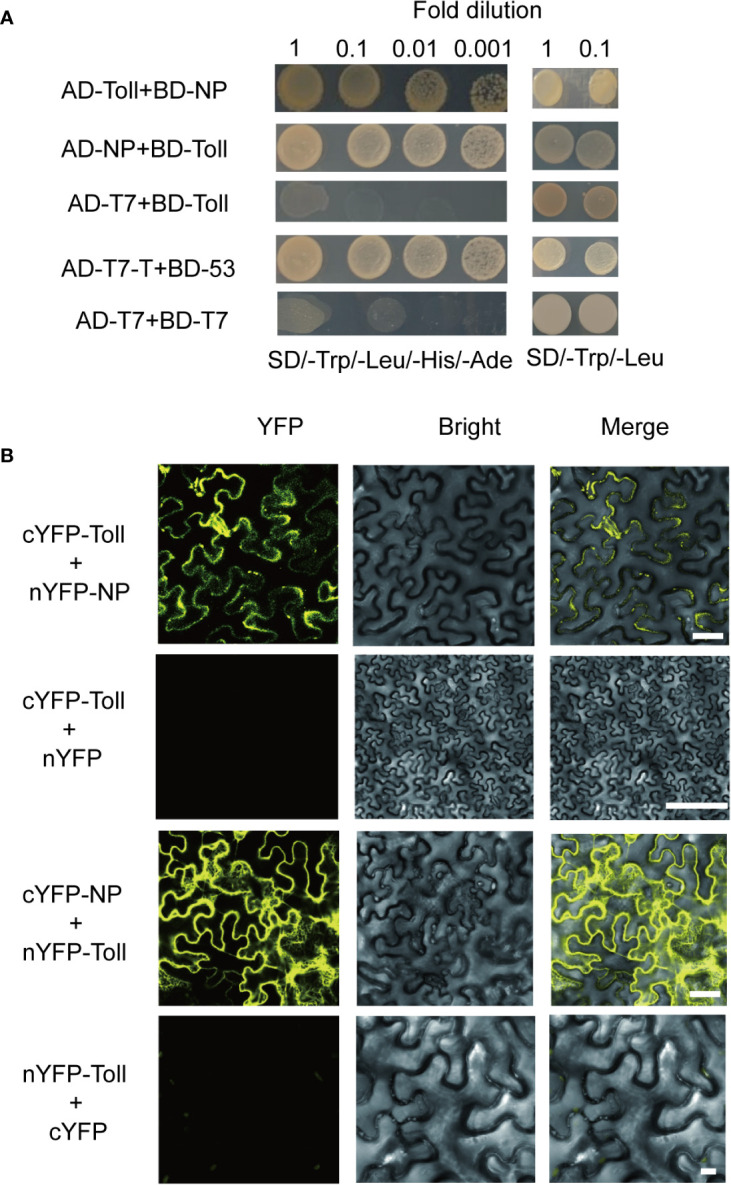
Protein-protein interaction analysis of Toll and rice stripe virus (RSV)-NP. **(A)** Yeast two-hybrid assay result showed that Toll interacted with RSV-NP protein in SD/–Leu/–Trp/–His/–Ade medium. **(B)** Bimolecular fluorescence complementation assays showed that pCV-cYFP–*Toll* and pCV–nYFP–*RSV-NP*, pCV–cYFP–*RSV*–*NP* and pCV–nYFP–*Toll* fluorescent strong YFP signals in the cytomembrane but there were no detectable signals in the negative control combinations pCV-cYFP–*Toll* and pCV–nYFP, pCV–cYFP, and pCV–nYFP–*Toll*. Bars, 50 µm.

To investigate the potential interaction between Toll and RSV proteins, seven viral proteins (NS2, NSvc2-C, NSvc2-N, NS3, NP, NS4, and MP) were used as baits to screen against the *L. striatellus* Toll. We found that Toll interacted with RSV-NP protein, but not the other six RSV proteins, and similar results were found when Toll was used as a bait and RSV-NP as a prey (**Figure 2A**, and **Figure S1**). In addition, yeast two-hybrid assay result showed that Toll could not interact directly with SPZ family including SPZ1, SPZ2, SPZ3, SPZ4, SPZ5, and SPZ6 proteins in SD/–Leu/–Trp/–His/–Ade medium (**Figure S2**). To confirm the interaction between planthopper Toll and RSV-NP, bimolecular fluorescence complementation (BiFC) assays were further performed in *N. benthamiana*. When pCV-cYFP-*Toll* and pCV-nYFP*-NP* were transiently co-expressed in *N. benthamiana* leaves, strong YFP fluorescence signals were observed in the cytomembrane, whereas no visible signal was detected in the negative control of pCV-cYFP–*Toll* and pCV–nYFP (**Figure 2B**). Similar results were found when pCV-cYFP-*NP* and pCV-nYFP*-Toll* were transiently co-expressed (**Figure 2B**). These results indicated that Toll and RSV-NP proteins interact directly and the *L. striatellus* Toll might act as PRR in recognizing signaling molecules of pathogen.

### Temporal and Spatial Expression of *Laodelphax striatellus* Toll

**Figure 3:**
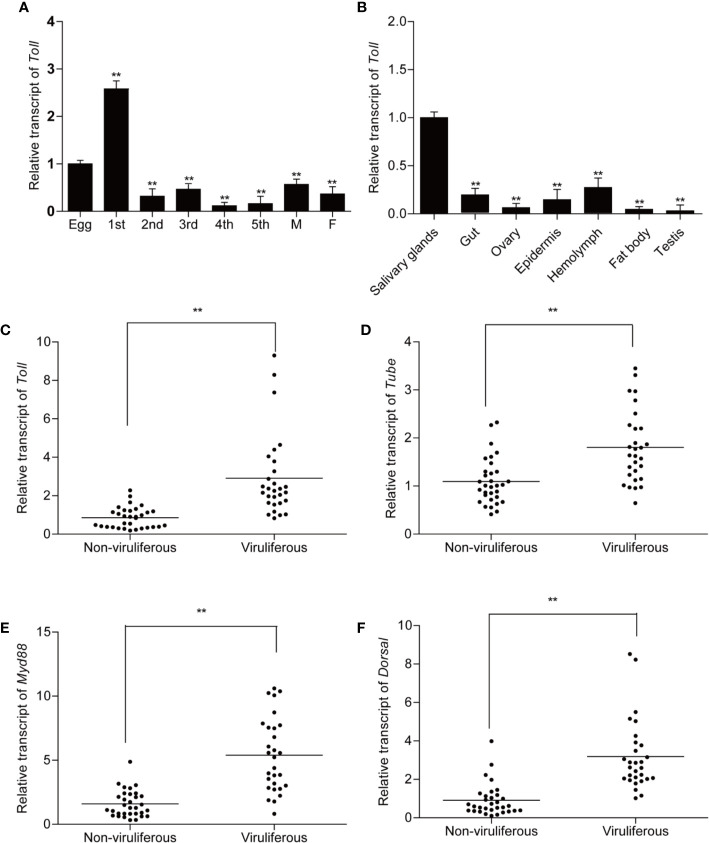
Expression patterns of *Toll* and relative transcript levels of *Toll*, *Tube*, *MyD88*, and *Dorsal* in non-viruliferous and viruliferous planthopper. For qPCR detection of *Toll*, samples from different developmental stages (eggs, nymphs from 1^st^ to 5^th^ instars, female and male adults) **(A)** and different tissues (salivary gland, gut, ovary, epidermis, hemolymph, fat body, and testicle) **(B)** were collected from non-viruliferous planthoppers. Five biological replicates were performed. For analysis of relative transcript levels of *Toll* **(C)**, *Tube* **(D, E)** *MyD88* **(E)**, and *Dorsal* **(F)**, non-viruliferous and viruliferous planthopper samples were collected individually. Actin gene was used as housekeeping gene. Each point represents a biological replicate. Statistically significant differences at *P* < 0.01 (**) level are indicated according to one-way analysis of variance (ANOVA) test.

To explore the expression pattern of Toll receptors, non-viruliferous planthopper samples from eight developmental stages and seven tissues were collected and quantified by qPCR. The results showed that *Toll* was ubiquitously expressed in all collected developmental stages and tissues of *L. striatellus* (**Figures 3A, B**). Messenger RNA (mRNA) of Toll was most abundant in the first instar nymphs of non-viruliferous planthopper, followed by eggs (**Figure 3A**). Furthermore, highest expression of *Toll* was observed in salivary glands compared with the other tissues of non-viruliferous planthoppers (**Figure 3B**).

### Active Response of the Canonical Toll Pathway During Rice Stripe Virus Infection

To illustrate the potential roles of Toll signaling pathway in RSV infection, the expressions of *Toll*, *Tube*, *MyD88*, and *Dorsal* were compared between viruliferous planthopper and non-viruliferous planthopper. Significantly increased expressions of all the four genes were observed in viruliferous planthopper population (**Figures 3C–F**), suggesting that Toll pathway might be actively involved in the stable maintenance of RSV in planthopper.

**Figure 4:**
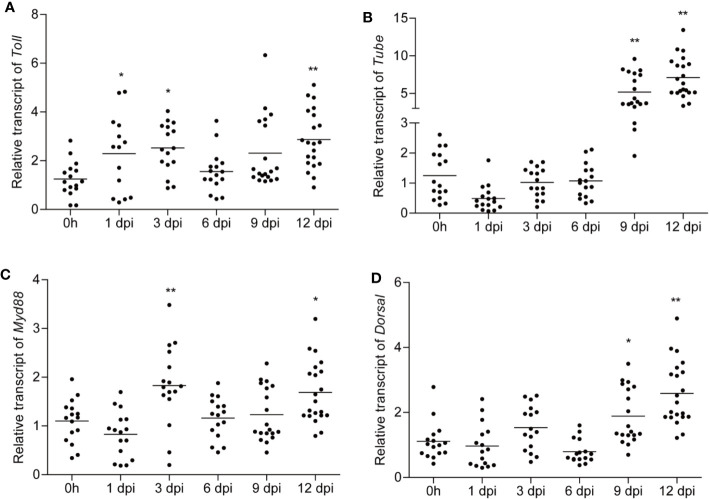
The expression pattern of *Toll*, *Tube*, *MyD88*, and *Dorsal* when non-viruliferous planthoppers were infected with rice stripe virus (RSV). Non-viruliferous planthoppers were fed on RSV-infected rice seedlings, and the samples were collected at 1, 3, 6, 9, and 12 days post-infection (dpi). Relative transcript levels of *Toll* **(A)**, *Tube* **(B)**, *MyD88* **(C)**, and *Dorsal* **(D)** at the indicated time points were analyzed by qPCR. The non-viruliferous planthopper that did not contact with RSV was used as a control. Actin gene was used as housekeeping gene. Each point represents a biological replicate. Statistically significant differences at *P* < 0.05 (*) and *P* < 0.01 (**) level are indicated according to one-way ANOVA test.

Moreover, previous studies demonstrated that virus infection activated the Toll pathway within a short period (16). Considering the remarkable upregulation of *Toll*, *Tube*, *MyD88*, and *Dorsal* in viruliferous planthopper, how the Toll pathway of non-viruliferous planthoppers responded to RSV infection is of interest. As a result, *Toll*, *Tube*, and *MyD88*, but not *Dorsal*, were actively responded during early stage of RSV infection (**Figure 4**). The expressions of *MyD88* and *Toll* were significantly increased after 1 and 3 days post-infection (dpi) (**Figures 4A, C**), whereas *Tube* was notably decreased after 1 dpi compare to that of the control (0 dpi) (**Figure 4B**). No significant change was detected after 6 dpi of RSV infection for both *Toll*, *Tube*, and *MyD88*. However, all of the four Toll pathway core genes were up-regulated after 9 and 12 dpi (at the late stage) of RSV infection (**Figure 4**). These dynamic expressions of *Toll*, *Tube*, *MyD88*, and *dorsal* imply the active and complexed involvement of the canonical Toll signaling pathway in response to the infection process of RSV.

### Potential roles of the Toll Pathway in the Maintenance of Rice Stripe Virus Proliferation

**Figure 5:**
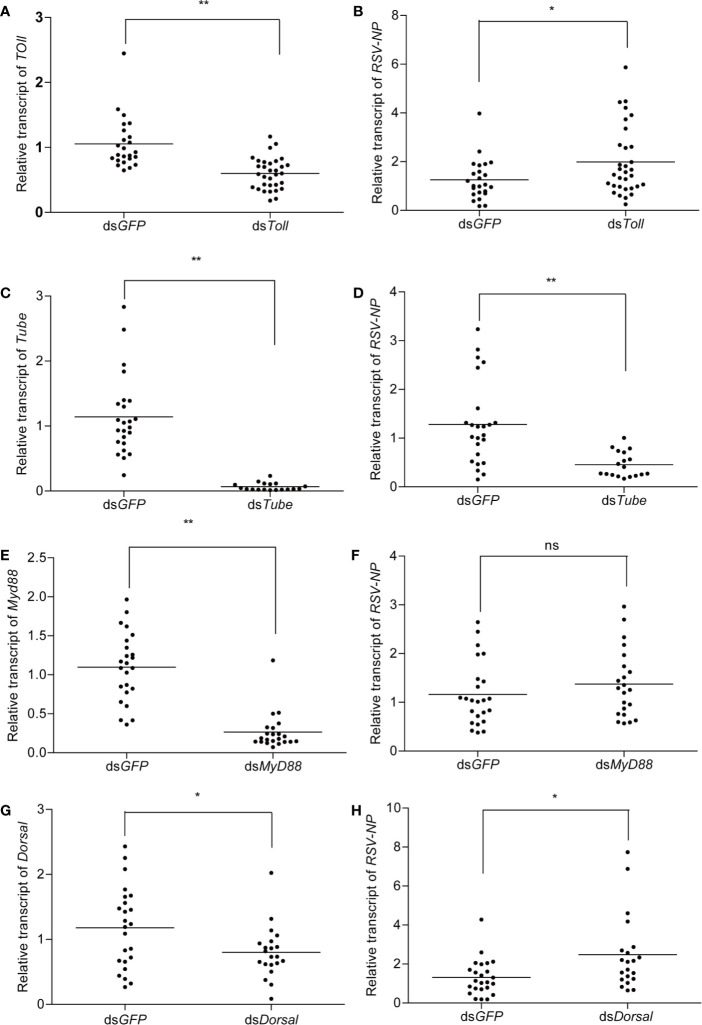
The influence of double-stranded RNA (dsRNA) treatment on viruliferous planthoppers. Approximately 25 viruliferous planthopper were injected with ds*Toll*, ds*Tube*, ds*MyD88*, and ds*Dorsal.* The silencing efficiency of *Toll* **(A)**, *Tube* **(C)**, *MyD88* **(E)**, and *Dorsal* **(G)** were determined. Meanwhile, the relative transcript level of RSV-NP after silencing of *Toll* **(B)**, *Tube* **(D)**, *MyD88* **(F)**, and *Dorsal* **(H)** were analyzed by qPCR. Planthopers treated with ds*GFP* were used as a negative control. Each point represents a biological replicate. Statistically significant differences at *P* < 0.05 (*) and *P* < 0.01 (**) level are indicated according to one-way ANOVA test. ns, not significant.

To investigate the potential roles of *Toll*, *Tube*, *MyD88*, and *Dorsal* in RSV infected planthoppers, dsRNA fragments corresponding to these four genes were synthesized and injected into the viruliferous planthoppers. Assessment of silencing efficient indicated the significant transcripts reduction (70%) for all of the four genes after 2 dpi (**Figures 5A, C, E, G**). Meanwhile, significant increase in titer of RSV was observed in ds*Toll-* and ds*Dorsal-*treated planthoppers (**Figures 5B, H**), whereas RNA level of RSV-NP was notably reduced for ds*Tube*-injected insects when compared with that of the control (ds*GFP*) (**Figure 5D**). In contrast, no significant difference was detected in RSV-NP between ds*MyD88* and ds*GFP-*treated planthoppers (**Figure 5F**).

### The Toll Pathway Is Involved in the Anti-Rice Stripe Virus Defense

**Figure 6:**
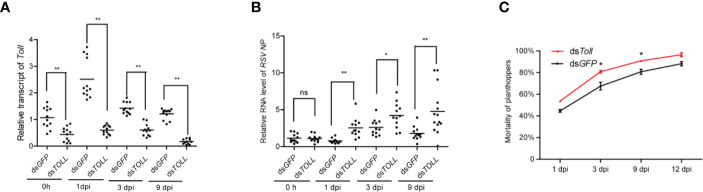
The influence of double-stranded RNA (dsRNA) treatment on non-viruliferous planthoppers that orally infected with rice stripe virus (RSV). The non-viruliferous planthopper were treated with ds*Toll* or ds*GFP.* Two days later, the insects were provided with RSV-infected rice seedlings, and the RNAi efficiency **(A)** and the amount of RSV in vector insects **(B)** were determined by qPCR at the indicated time point. Each point represents a biological replicate. **(C)** The mortality of RSV-infected planthopper after ds*Toll* and ds*GFP* treatments. Three biological replicates were performed. Statistically significant differences at *P* < 0.05 (*) and *P* < 0.01 (**) level are indicated according to one-way ANOVA test. ns, not significant.

In view of the direct interaction between Toll and RSV-NP, the accumulation of RSV-NP transcripts was further examined in ds*Toll*-treated planthoppers when non-viruliferous planthoppers were infected by RSV. Injection of ds*Toll* successfully and stably inhibits the *Toll* expression in planthoppers after RSV acquisition for various days (**Figure 6A**). The effects of ds*Toll*-injection on the RSV proliferation in planthoppers were further determined. Significant increase in RSV-NP transcripts were observed in ds*Toll* injected planthopper compared to that of the control (ds*GFP*) at various infected time point (1, 3, and 9 dpi) (**Figure 6B**). Additionally, the mortality of RSV-infected planthopper was significantly higher after ds*Toll* treatment in 3 and 9 dpi than that of ds*GFP* control (**Figure 6C**). These data suggested that Toll might play an essential role in restricting RSV proliferation.

## Discussion

Accumulated evidence demonstrated that the innate immune system plays an important role in defense against viruses in mammal and some model insects such as *Drosophila* and mosquitoes (1, 13–16). However, whether the canonical pathway of vector insects also involved in defense against plant viruses remained unknown. In this study, we found that RSV activated the Toll immune pathway of *L. striatellus* through direct interaction between Toll protein and RSV-NP. Knockdown of *Toll* significantly increased the proliferation of RSV in vector insect, and the ds*Toll*-treated insects exhibited higher mortality than that of ds*GFP*-treated ones. Our results suggested a potential role of Toll pathway in restrict plant virus infection.

Activation of immune pathways relies on an array of PRRs to recognize the PAMPs, and subsequently induce an appropriate effector response to clear the infection (36). For Toll pathway, this process was mainly accomplished by Toll, which is the upstream receptor of this pathway. In *Drosophila*, Toll-7 is a PRR that interacted with VSV at the plasma membrane and induced antiviral autophagy (2). In shrimp, knockdown of Toll4 results in elevated viral loads and renders shrimp more susceptible to WSSV infection. Furthermore, Toll4 could act as an upstream PRR to detect WSSV, and lead to nuclear translocation and phosphorylation of Dorsal for the trigger of AMP production against the virus (16). Our study identified a strong interaction between Toll and RSV-NP, indicating that Toll in *L. striatellus* might be an upstream receptor to recognize RSV, and Toll pathway was associated with plant virus infection in insect vector.

Toll pathways is the major constitute of insect immune pathways that activate a battery of immune proteins in response to various microorganism invasion. Remarkable upregulation in Toll pathway genes were reported in *Aedes aegypti* challenged with *Plasmodium gallinaceum* (37), *Drosophila* challenged with Vesicular stomatitis virus (2), and *Litopenaeus vannamei* challenged with WSSV (16). Our study demonstrated that *Toll*, *Tube*, and *MyD88* were actively responded during early stage of RSV oral infection (**Figure 4**), in accordance of previous work. For the transcription factor *Dorsal*, it is stable expressed at the early stage of viral infection, but significantly upregulated at the late stage (**Figure 4**). Since *Toll*, *Tube*, *MyD88*, and *Dorsal* are the four core genes of the canonical Toll-Dorsal signaling pathway, the upregulation of these four genes at various stages during RSV infection (**Figure 4**), the interaction between Toll and RSV-NP (**Figure 2**), as well as the increased viral titers observed in ds*Toll*-treated planthoppers (**Figure 6**), implying that this classical pathway was actively involved in response to RSV infection. Nevertheless, more studies are needed to further investigate on the detail of downstream antiviral response, such as how does Dorsal translocate from cytoplasm into the nucleus, and which downstream effectors are regulated by Dorsal induced with RSV infection. In addition, for viruliferous planthopper, they can harbor the viruses for several generations, and no significant phenotype can be found in the RSV-infected insects. In this study, it is interesting to find that the expression level of four Toll pathway core genes were significantly higher in viruliferous planthopper than that in non-viruliferous one (**Figure 3**). Sustained activation of defense pathway inevitably consumes extra resources, which is detrimental to insects (38). We presumed that it might be more important for planthoppers to restrict RSV infection than other physiological metabolisms. Interestingly, higher mortality rate was recognized in ds*Toll*-treated viruliferous planthoppers (**Figure 6C**), suggesting that *dsToll*-treatment might interfere with the established delicate balance between innate immunity of planthopper and persistent RSV infection, as described in mosquitoes (39). Our results also consist with the previous report that TLR4 knockdown mice exhibited greater viral replication (Vaccinia virus) and mortality compared to the wild-type mice following respiratory infection (40), indicating that the Toll signaling pathway of the host might be essential for the virus persistent infection.

Involvement of Toll pathway in restrict virus infection has been well documented in previous work. In *Drosophila*, *Toll* and *Dif* mutant lines showed increased susceptibility to *Drosophila* X virus (1), and *Toll-7* depletion promoted vesicular stomatitis virus replication (2). In shrimps, silencing of *Toll-4* resulted in high WSSV titers, with the average viral DNA burden approximately 150 times higher than that of the control (16). In *A. aegypti*, silencing of *MyD88* led to a significant increase in dengue virus titers, demonstrating the importance of this innate immune pathway in the defense against different dengue virus serotypes at the early stages of infection (13). Our study demonstrated that *Toll-*inhibition and *Dorsal*-inhibition significantly increased the RSV titer, suggesting the potential antiviral roles of Toll pathway against plant virus. However, for ds*MyD88*-treatment viruliferous planthoppers, no significant change in RSV titer was observed when compared to the control (ds*GFP*) (**Figure 5F**), which is inconsistent with the previous studies in mosquitoes (13) and mice (41). Considering the increased expression of *MyD88* in response to RSV infection (**Figure 4C**), we presume that *MyD88* might play more important roles during the process of RSV infection, rather than the maintenance of RSV persistent infection in planthoppers. Furthermore, unexpectedly, RSV titer in ds*Tube*-treatment was significantly decreased compare to control (ds*GFP*) (**Figure 5D**). It will be interesting to further explore the possibility that whether Tube can interact directly with the protein of RSV and might be hijacked by the virus to promote its proliferation.

## Conclusion

In summary, we found that Toll pathway was activated upon RSV infection, and the viruliferous planthopper exhibited higher level of *Toll*, *Tube*, *MyD88*, and *Dorsal*. More intriguing, unlike the classical Toll signaling pathway which rely on the Spz binding to the Toll receptor, our study provide the first evidence that the antiviral Toll signaling pathway of *L. striatellus* is potentially activated through the direct interaction between Toll receptor and PAMPs (RSV-NP), suggesting that Toll immune pathway is an important strategy against plant viruses in insect vectors.

## Data Availability Statement

The datasets presented in this study can be found in online repositories. The names of the repository/repositories and accession number(s) can be found below: https://www.ncbi.nlm.nih.gov/genbank/, MW048393 https://www.ncbi.nlm.nih.gov/genbank/, MW048395 https://www.ncbi.nlm.nih.gov/genbank/, MW048396 https://www.ncbi.nlm.nih.gov/genbank/, MW048394.

## Author Contributions

J-ML, J-PC, and C-XZ conceived the study and designed the project. Y-JH, GL, YZ, Y-HQ, Z-TS, X-DZ, and J-CZ performed the experiment, analyzed the data, and drafted the manuscript. H-JH, FY, and J-ML helped to revise the manuscript. All authors contributed to the article and approved the submitted version.

## Funding

Present research was financially supported by the National Natural Science Foundation of China (32000121), Ningbo Science and Technology Innovation 2025 Major Project (2019B10004) and Commonweal Project (202002N3004, 202002N3008). This work was sponsored by K.C. Wong Magna Fund in Ningbo University.

## Conflict of Interest

The authors declare that the research was conducted in the absence of any commercial or financial relationships that could be construed as a potential conflict of interest.

## Supplementary Material

Supplementary Figure 1 Protein-protein interaction between Toll and RSV NS2, NSvc2-C, NSvc2-N, NS3, NS4, and MP.

Supplementary Figure 2 Protein-protein interaction between Toll and SPZ family of planthopper.
