# Supplementary figures and images for: Activation of Toll Immune Pathway in an Insect Vector Induced by a Plant Virus
Source: Front Immunol. 2021 Jan 8;11:613957. doi: 10.3389/fimmu.2020.613957 (PMC7821435; doi:10.3389/fimmu.2020.613957)

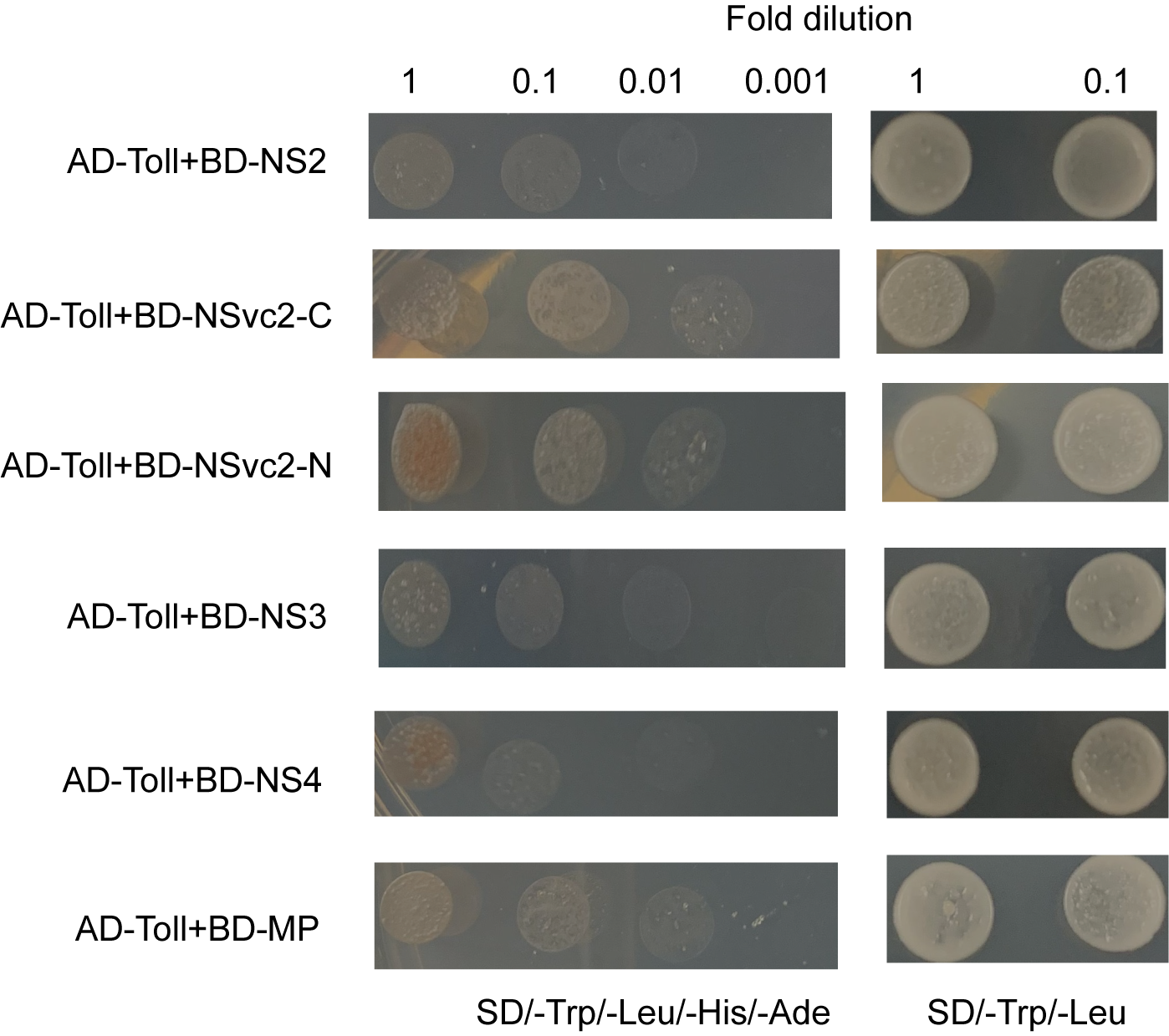

Supplement: Supplementary Figure 1 — Protein-protein interaction between Toll and RSV NS2, NSvc2-C, NSvc2-N, NS3, NS4, and MP. [file Image_1.tif]

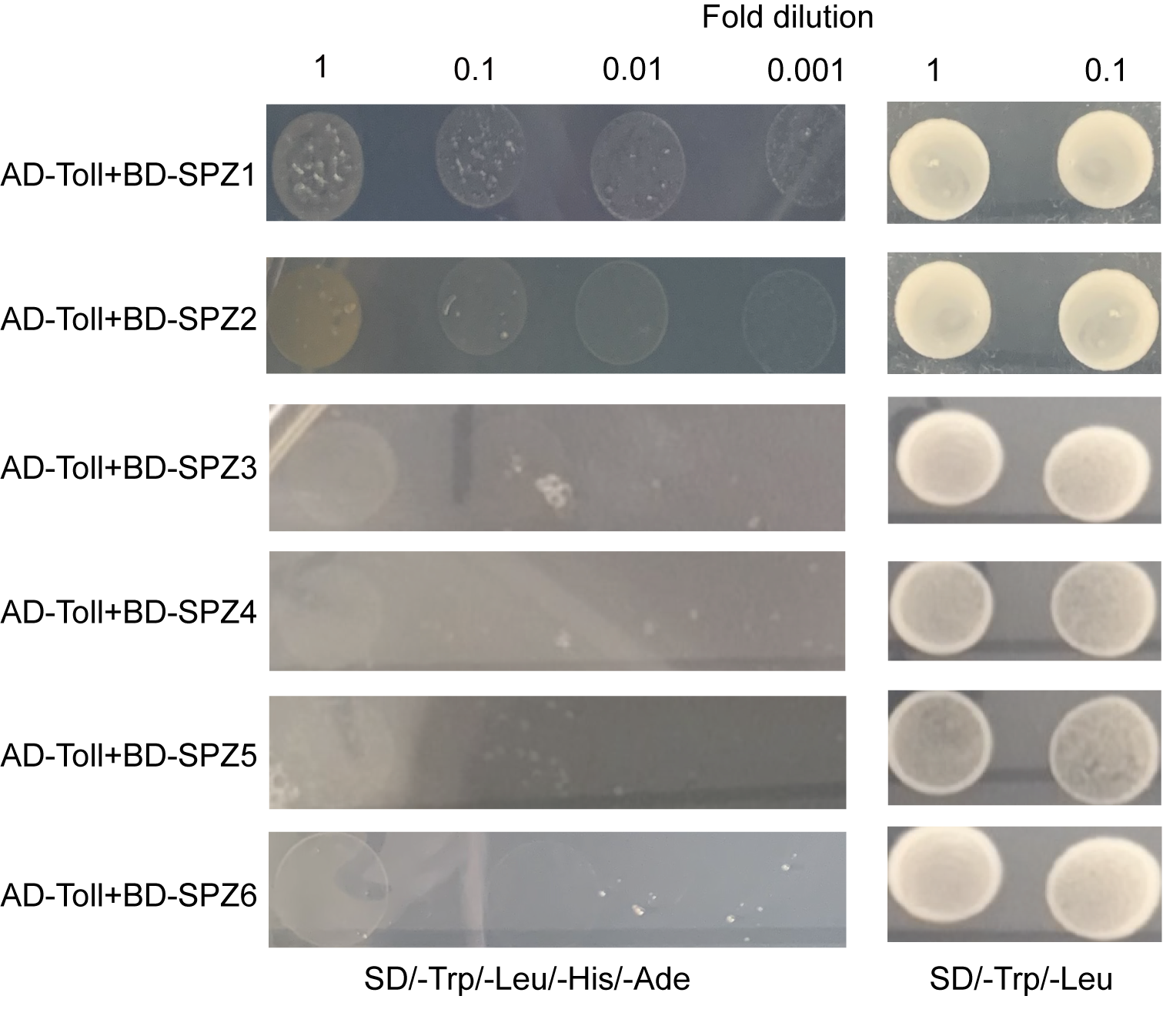

Supplement: Supplementary Figure 2 — Protein-protein interaction between Toll and SPZ family of planthopper. [file Image_2.tif]
