# Supplementary material for: Activation of Toll Immune Pathway in an Insect Vector Induced by a Plant Virus
Source: Front Immunol. 2021 Jan 8;11:613957. doi: 10.3389/fimmu.2020.613957 (PMC7821435; doi:10.3389/fimmu.2020.613957)
Supplement: Supplementary file 3 [file Table_1.docx]

**Table S1 Primers used in this study.**

| Primer names | Sequence ( 5’-3’) |
| --- | --- |
| **Gene clone** |  |
| Toll-F | ATGTGCAAGGAGAAGATGCC |
| Toll-R | CTATACTTTGACTTGTGCTT TG |
| Tube-F | ATGTCTGTAGATACAGAATTGA |
| Tube-R | TTATAAAGGTATTCTTCTTGAGA |
| Myd88-F | ATGGCAGCTACTGAGGTGG |
| Myd88-R | TTAAGCTGGGACAGGCAGT |
| Dorsal-F | ATGGACACGGACATTGAAGC |
| Dorsal-R | CTATTTAACA GGAACATTGCTA |
|  | |
| **Tissue distribution and expression pattern analysis** | |
| Q-Toll-F | AACCCTCTCAACTGCGACTG |
| Q-Toll-R | GTGACGGAATCGTGGTCACT |
| Q-Tube-F | TTCCACTGGTGACAGTTCCG |
| Q-Tube-R | TATCAATTCGCCGTGTCGGT |
| Q-Myd88-F | AGGTGGCGGAAAGATCAACT |
| Q-Myd88-R | GGGTGTCGGAACATCACAAAG |
| Q-Dorsal-F | TGCCCAGCAAAGACCTACTG |
| Q-Dorsal-R | TGCTAACACCGGGAATGCTT |
| Q-Actin-F  Q-Actin-R | AGTGCCCATCTACGAAGGTTACG  CGGCGGTGGTGGTGAAGC |
|  |  |
| **RNA interference** |  |
| Toll-RNAi-F | TAATACGACTCACTATAGGGAGATTTGATGACGTCGATGGGCA |
| Toll-RNAi-R | TAATACGACTCACTATAGGGAGACCAATTTGCTGAGATCGGCG |
| Tube-RNAi-F | TAATACGACTCACTATAGGGAGATCTGAGCGCTGCTACTGATG |
| Tube-RNAi-R | TAATACGACTCACTATAGGGAGACGTACCAAACCGAAATCGCC |
| Myd88-RNAi-F | TAATACGACTCACTATAGGGAGACTGGAACAGTTGGCGAAAAGG |
| Myd88-RNAi-R | TAATACGACTCACTATAGGGAGAGCGCTTGAGCGAAATTCGTA |
| Dorsal-RNAi-F | TAATACGACTCACTATAGGGAGAATCAGCCAGGTAGCATCGAC |
| Dorsal-RNAi-R | TAATACGACTCACTATAGGGAGAAGCTGATTGAATGGGCGTCA |
| GFP-RNAi-F | TAATACGACTCACTATAGGGAGTGGAGAGGGTGAAGG |
| GFP-RNAi-R | TAATACGACTCACTATAGGGAGGGCAGATTGTGTGGAC |
